# Time-Dependent Changes of Klotho and FGF-23 Levels after Kidney Transplantation: Role of Cold Ischemia Time, Renal Function and Graft Inflammation

**DOI:** 10.3390/jcm12134486

**Published:** 2023-07-04

**Authors:** Teresa Vazquez-Sanchez, Maria Dolores Sanchez-Niño, Pedro Ruiz-Esteban, Veronica López, Myriam León, Abelardo Caballero, Juan Francisco Ruiz-Escalera, Alberto Ortiz, Armando Torres, Mariano Rodriguez, Domingo Hernandez

**Affiliations:** 1 Nephrology Department, Hospital Universitario Regional de Málaga, Universidad de Málaga, Instituto de Investigación Biomédica de Málaga (IBIMA)-Plataforma BIONAND, RICORS2040 (RD21/0005/0012), E-29010 Malaga, Spain; teresavs89@hotmail.com (T.V.-S.); pedro_ruiz_esteban@hotmail.com (P.R.-E.); verolopezjim@yahoo.es (V.L.); 2 Pharmacology Department, IIS-Fundacion Jimenez Diaz, Universidad Autonoma de Madrid, RICORS2040 (RD21/0005/0001), E-28040 Madrid, Spain; mdsanchez@fjd.es; 3 Pathology Department, Hospital Universitario Regional de Málaga, Universidad de Málaga, Instituto de Investigación Biomédica de Málaga (IBIMA)-Plataforma BIONAND, RICORS2040 (RD21/0005/0012), E-29010 Málaga, Spain; mlfradejas@gmail.com; 4 Immunology Department, Hospital Universitario Regional de Málaga, Universidad de Málaga, Instituto de Investigación Biomédica de Málaga (IBIMA)-Plataforma BIONAND, RICORS2040 (RD21/0005/0012), E-29010 Malaga, Spain; abelardo.caballero.sspa@juntadeandalucia.es; 5 Clinical Analysis Department, Hospital Universitario Regional de Málaga, Universidad de Málaga, Instituto de Investigación Biomédica de Málaga (IBIMA)-Plataforma BIONAND, RICORS2040 (RD21/0005/0012), E-29010 Malaga, Spain; juanf.ruiz.escalera.sspa@juntadeandalucia.es; 6 Nephrology Department, IIS-Fundacion Jimenez Diaz, Universidad Autonoma de Madrid, RICORS2040 (RD21/0005/0001), E-28040 Madrid, Spain; aortiz@fjd.es; 7 Nephrology Department, Hospital Universitario de Canarias, Instituto de Tecnologías Biomédicas, Universidad La Laguna, REDinREN (RD16/0009/0031), E-38320 Tenerife, Spain; atorresram@gmail.com; 8 Nephrology Department, Maimonides Institute for Biomedical Research of Cordoba (IMIBIC), Reina Sofia University Hospital, University of Cordoba, RICORS2040 (RD21/0005/0008), E-14004 Cordoba, Spain; marianorodriguezportillo@gmail.com

**Keywords:** kidney transplant, serum klotho levels, serum FGF-23 levels, graft function, graft subclinical inflammation, cold ischemia time

## Abstract

We investigated the evolution of serum klotho (s-Kl) and FGF-23 during the first two years post-kidney transplantation (KT), considering the cold ischemia time (CIT), glomerular filtration rate (GFR) and graft subclinical inflammation (SCI). We undertook a prospective, cohort, multicenter study of consecutive patients between April 2018 and January 2021 (with follow-up at 24 months). Subgroups were analyzed according to the median CIT (<14 vs. ≥14 h), the median GFR (≤40 vs. >40 mL/min/1.73 m^2^) and the presence of SCI at month 3. A total of 147 patients were included. s-Kl and fibroblast growth factor-23 (FGF-23) levels were measured at baseline and at months 3, 12 and 24. Graft biopsies (*n* = 96) were performed at month 3. All patients had low s-Kl levels at month 3. Patients with CIT < 14 h exhibited a significant increase in s-Kl at month 24. In patients with CIT ≥ 14 h, s-Kl at month 3 fell and lower s-Kl levels were seen at month 24. Patients with a GFR > 40 had a lesser decrease in s-Kl at month 3. FGF-23 fell significantly at months 3 and 12 in both GFR groups, a reduction maintained during follow-up. There were significant inter-group differences in s-Kl from months 3 to 24. CIT, GFR at 3 months and SCI were significantly associated with s-KI at month 3. A reduction in s-Kl at month 3 post-KT could be explained by longer CIT and delayed graft function as well as by impaired graft function. Early SCI may regulate s-Kl increase post-KT.

## 1. Introduction

Abnormal mineral bone metabolism is universal in patients with chronic kidney disease (CKD). Serum klotho (s-Kl), resulting from the cleavage of membrane-bound klotho, is significantly reduced in CKD patients because the kidneys are the major source of klotho, the co-receptor of the phosphaturic hormone fibroblast growth factor-23 (FGF-23). s-Kl has extra-renal effects beyond mineral metabolism, including antioxidative and antifibrotic properties [1].

Kidney transplantation (KT) partially restores CKD-associated disorders. Indeed, the serum concentration of s-Kl increases significantly at 4–13 months after KT and FGF-23 is reduced after KT [2,3]. However, a sustained rise in s-Kl levels has not been conclusively proven post-KT, and there is a paucity of data assessing time-dependent changes of s-KI and FGF-23 beyond the first year after KT. In addition, information is lacking regarding the association of s-Kl and FGF-23 with clinical parameters inherent to KT, such as allograft function or cold ischemia time (CIT).

Early graft subclinical inflammation (SCI) is very prevalent post-KT, even with proper immunosuppression in low-immunological risk KT recipients. Klotho expression may be regulated by inflammatory cytokines [4] and s-Kl has been reported as an anti-inflammatory molecule [5,6]. However, there is a lack of information about the relationship between early SCI and s-Kl levels when protocol biopsies (PB) are routinely performed in these patients.

To our knowledge, only a few prospective studies have evaluated the changes in s-Kl and FGF-23 levels during the first year post-KT and no longitudinal studies beyond the first 12 months have assessed the relationship between these molecules and renal graft function [7,8,9]. We hypothesized that a more prolonged CIT, an impaired graft function or the presence of SCI could modify s-Kl and FGF-23 levels following KT.

The aim of this longitudinal study was to assess s-Kl and FGF-23 levels in incident deceased-donor KT patients during the first two years post-KT and to determine their relationship with CIT and allograft function.

## 2. Materials and Methods

### 2.1. Study Design

This was a prospective, cohort, multicenter study of consecutive patients, conducted between April 2018 and January 2021 in the Nephrology Department of the Regional University Hospital of Málaga. Patients of both sexes over 18 years of age who signed the informed consent for participation in the study were included. Those who received a double transplant (renal–pancreatic) were excluded. All patients were followed up for 24 months.

The study was undertaken in accordance with the standards of good clinical practice and the ethical concepts of the Declaration of Helsinki.

### 2.2. Data Collection

The clinical data were acquired by staff after reviewing the medical history. Graft function was estimated by the 2009 creatinine-based CKD-EPI formulae for estimated glomerular filtration rate (eGFR). The following analytical parameters were collected pre-KT and 3, 12 and 24 months post-KT: glycemia, HbA1c, total cholesterol, high-density lipoprotein (HDL) cholesterol, low-density lipoprotein (LDL) cholesterol, triglycerides, calcium, phosphate, fractional excretion phosphorus, parathyroid hormone and proteinuria. Other molecules, tumor necrosis factor (TNF) and tumor necrosis factor-like weak inducer of apoptosis (TWEAK) levels were also determined following the manufacturer’s instructions. Other data collected included cold ischemia time, acute rejection, induction therapy, delayed graft function (DGF), end-stage renal disease, post-transplantation diabetes mellitus, panel-reactive antibodies, donor and recipient ages, expanded criteria donor, gender, hemodialysis, body mass index, cardiovascular disease and immunosuppressants by intention to treat.

### 2.3. Measurements of Serum Klotho and FGF-23

Levels were measured just before KT and at 3, 12 and 24 months post-KT. Blood samples were collected, processed and frozen at −80 °C within 2 h of extraction. s-Kl levels were determined at the Nephrology and Hypertension Laboratory of the IIS-Fundación Jiménez Díaz (Madrid, Spain) using a specific ELISA kit for serum human klotho (ELISA, Immuno-Biological Labs, Fujioka-Shi, Japan) following the manufacturer’s instructions. The coefficient of variability (%CV) was 2.7–6.5% and the limit of detection was 6.15 pg/mL. Serum levels of FGF-23 were determined at the Immunology Laboratory of the Biometric Research Institute of Málaga, Regional University Hospital of Málaga, using a specific ELISA kit (ELISA, Kainos Laboratories, Tokyo, Japan) following the manufacturer’s instructions. This assay had an inter- and intra-assay coefficient of variation of 2.1 to 3.8% and 2.0 to 3.0%, respectively, and a low limit of detection of 3 pg/mL.

### 2.4. Histological Data

Protocol biopsies were performed at 3 months post-transplant in 97 KT recipients as an outpatient procedure and were carried out under ultrasound guidance using an 18 G spring-loaded biopsy needle. At least one core of tissue with a minimum of seven glomeruli and one artery were required for proper interpretation. Graft SCI included either the presence of borderline lesions defined as an interstitial inflammation score (i) and/or tubulitis score (t) of at least 1, but below the threshold for Banff 1A rejection (Banff i2, t2) [10], or a higher degree of graft inflammation according to the 2019 Banff classification [11]. Neither isolated inflammation (i1, t0) nor tubulitis (i0, t1) were included as SCI. Additionally, the chronic allograft histology score was obtained using a composite of chronic interstitial (ci), chronic tubular (ct), chronic glomerular (cg) plus chronic vascular (cv) (ci + ct + cg + cv), as well as using a composite of interstitial fibrosis and tubular atrophy (IFTA) score (ct + ci). IFTA was defined as the sum of ci + ct ≥ 2 [12,13]. Experienced transplant pathologists interpreted all biopsies and inflammatory and chronicity scores were all validated by a single pathologist (ML).

### 2.5. Statistical Analysis

A descriptive analysis of the results was undertaken, expressing the quantitative variables as the mean ± standard deviation for parametric data or median and interquartile range (IQR) for non-parametric data. Categorical variables were expressed as numbers and percentages. For univariate comparisons of numerical variables, the *t*-test and chi-square test were used. Correlations between biochemical parameters (s-Kl, FGF-23 and GFR) were performed by univariate regression. We performed multivariate linear regression analysis of factors associated with s-Kl at three months post-transplant. A repeated-measures ANOVA model was used to evaluate pre- and post-KT differences in the s-Kl levels and FGF-23 levels over time.

Statistical analysis was performed with SPSS Statistics V26.0 for Windows (IBM Corp., Armonk, NY, USA) and the significance was set at *p* < 0.05.

## 3. Results

We included 147 recipients who received a KT from April 2018 to January 2021. Most patients received either basiliximab (*n* = 51) or thymoglobulin (*n* = 73) as induction therapy; maintenance immunosuppression consisted of steroids, calcineurin inhibitors and mycophenolate mofetil (Table 1). DGF was defined as the need for dialysis during the first week after KT.

Overall, a non-significant numerical reduction in s-Kl levels at month 3 was observed in the whole cohort. Thereafter, KT recipients were divided into subgroups according to the median CIT (≤14 vs. >14 h) and the median GFR at month 3 post-KT (≤40 vs. >40 mL/min/1.73 m^2^). No significant differences were found between CIT or eGFR groups at month 3 in s-Kl or FGF-23 levels, but a higher phosphaturia level was seen in patients with GFR > 40 mL/min/1.73 m^2^, as expected (Table 2 and Table 3). Finally, a significant correlation between GFR and FGF-23 was observed at months 3 (r = −0.263; *p* = 0.05) and 24 (r = −0.431; *p* = 0.001). Likewise, there was a significant correlation between GFR and s-Kl at month 24 (r = 0.260; *p* = 0.011).

**Figure 1:**
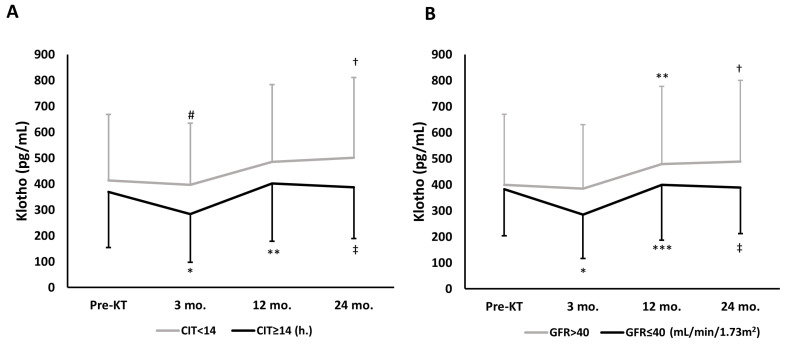
Overall time-dependent changes of s-Kl during follow-up, assessed by repeated-measures ANOVA model, according to CIT or GFR at month 3. (**A**) According to CIT < 14 h; *p* = 0.009, and CIT ≥ 14 h; *p* < 0.001. Intragroup comparisons * *p* = 0.023 vs. pre-KT; ** *p* < 0.001 vs. 3 mo.; ^†^ *p* = 0.028 vs. 3 mo.; ^‡^ *p* = 0.001 vs. 3 mo. Intergroup comparisons (CIT < 14 vs. ≥14 h): ^#^ *p* = 0.052 vs. 3 mo. CIT ≥ 14 h. group. (**B**) According to GFR values at month 3. GFR > 40 mL/min/1.73 m^2^; *p* = 0.002, and GFR ≤ 40 mL/min/1.73 m^2^; *p* < 0.001. Intragroup comparisons: * *p* = 0.006 vs. pre-KT; ** *p* = 0.019 vs. 3 mo.; *** *p* = 0.001 vs. 3 mo.; ^†^ *p* = 0.001 vs. 3 mo.; ^‡^ *p* = 0.008 vs. 3 mo. Abbreviations: CIT, cold ischemia time; GFR, glomerular filtration rate; KT, kidney transplant; s-Kl, serum klotho levels.

Patients with CIT < 14 h were less likely to have DGF than those with CIT ≥ 14 h (24.3% vs. 47.1%, respectively; *p* = 0.004) and exhibited a significant increase over time in s-Kl at month 24. In patients with CIT ≥ 14 h, s-Kl at month 3 fell when compared to pre-KT values (Figure 1A). Among patients with CIT ≥ 14 h, s-Kl levels below median values (324 pg/mL, interquartile range 182–496 pg/mL) were more common than s-Kl levels above s-Kl median levels (63.9 vs. 36.1%, respectively; *p* = 0.029).

In patients with a GFR ≤ 40 mL/min/1.73 m^2^ at month 3, s-Kl at month 3 fell when compared to pre-KT values. Interestingly, in patients with better graft function at month 3, s-Kl levels increased significantly and progressively at months 12 and 24, whereas in patients with a lower GFR, s-Kl levels increased at month 12, but no further at month 24 (Figure 1B).

**Figure 2:**
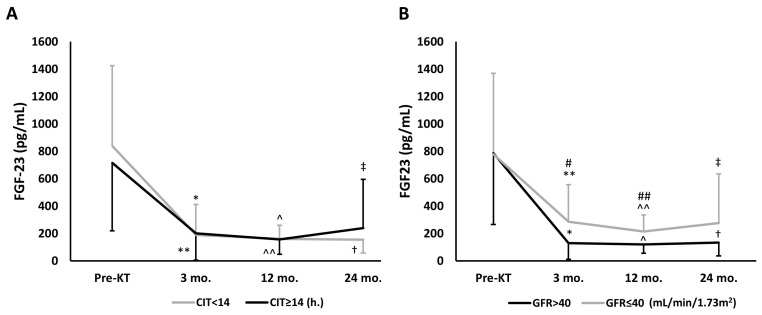
Overall time-dependent changes of FGF-23 during follow-up, assessed by repeated-measures ANOVA model, according to CIT or GFR. (**A**) According to CIT. CIT < 14 h; *p* = 0.009, and CIT ≥ 14 h.; *p* < 0.001. Intragroup comparisons: * *p* < 0.001 vs. Pre-KT; ** *p* < 0.001 vs. Pre-KT; ^ *p* < 0.001 vs. Pre-KT*;* ^^ *p* < 0.001 vs. Pre-KT*;* ^†^ *p* < 0.001 vs. Pre-KT; ^‡^ *p* < 0.001 vs. Pre-KT. (**B**) According to GFR. GFR > 40 mL/min/1.73 m^2^; *p* < 0.001, and GFR ≤ 40 mL/min/1.73 m^2^; *p* < 0.001. Intragroup comparisons: * *p* < 0.001 vs. Pre-KT; ** *p* < 0.001 vs. Pre-KT; ^ *p* < 0.001 vs Pre-KT; ^^ *p* < 0.001 vs. Pre-KT*;* ^†^ *p* < 0.001 vs. Pre-KT; ^‡^ *p* = 0.002 vs. Pre-KT. Intergroup comparisons (GFR > 40 vs. GFR ≤ 40 mL/min/1.73 m^2^): ^#^ *p* = 0.018 vs. 3 mo.; *^##^ p* = 0.003 vs. 12 mo. Abbreviations: CIT, cold ischemia time; FGF-23, fibroblast growth factor-23; GFR, glomerular filtration rate; KT, kidney transplant.

Figure 2 displays the time-dependent changes of FGF-23 during follow-up. FGF-23 fell significantly at month 3 regardless of CIT, with levels remaining unchanged during follow-up (Figure 2A), while FGF-23 dropped significantly at months 3 and 12 in both GFR groups, though there were significant differences between the groups from months 3 to 24, and this reduction was maintained in all patients during follow-up (Figure 2B).

Table 4 displays histological findings in protocol biopsies. Only 32 KT patients showed no SCI and borderline lesions were observed in 47.4% of the KT recipients. The other patients showed a higher degree of SCI. As expected, SCI patients presented significantly higher inflammation scores than KT recipients without inflammation despite similar tacrolimus levels in both groups. There were no statistically significant differences in s-Kl levels between SCI groups.

Finally, CIT, baseline s-Kl and GFR and SCI at month 3 correlated with s-Kl at month 3 in the multiple linear regression analysis after adjusting for confounders (Table 5). When SCI was substituted by the presence of a relevant interstitial inflammation score (i ≥ 1) or tubulitis score (t ≥ 1), again a significantly positive correlation with s-Kl at month 3 was observed in the linear regression analysis adjusted for variable confounders (i ≥ 1, Beta = 81.790; 18.834–144.746 95% CI; *p* = 0.012 and *t* ≥ 1, Beta = 90.601; 31.746–149.457 95% CI; *p* = 0.003).

## 4. Discussion

This study evaluated the time-dependent changes in biomarkers such as s-Kl and FGF-23 levels following KT. To our knowledge, this is the first prospective observational cohort study conducted in deceased-donor KT patients to determine changes in s-Kl and FGF-23 levels over the first two years post-KT and to assess their relationship with CIT, allograft function and SCI.

As hypothesized, s-Kl increased following KT, although notably only after month 3 post-KT. The s-Kl levels showed an initial reduction in the early post-KT period (month 3), which was significant in patients with lower GFR or longer CIT, followed by a gradual increase at 12 and 24 months post-KT, which was more pronounced in patients with better graft function or a shorter CIT. The explanation for this is likely multifactorial. Klotho is expressed mostly in distal renal tubules. Graft ischemia–reperfusion injury (IRI) downregulates klotho expression, which might be magnified by immunosuppressive load [7,14,15]. Thus, a transient decrease in s-Kl during the first three months is likely, as seen in our study. The fact that a higher proportion of DGF was observed in the CIT ≥ 14 h group and that CIT correlated with s-Kl levels during follow-up supports this argument.

Because most s-Kl is produced by the kidneys and correlations between GFR and s-Kl have already been documented, an increase in s-Kl levels after month 3 was not unexpected. In fact, s-Kl levels were numerically higher in the GFR > 40 mL/min/1.73 m^2^ group during follow-up as compared with those with a more reduced renal function. This is consistent with other observational studies and suggests that graft function could be a determinant for recovering circulating s-Kl levels after KT, although levels are still lower than in age-matched healthy controls. Two principal factors could be involved in s-Kl levels recovering after month 3: firstly, the restoration of klotho expression in the transplanted graft post-IRI, plus reduction of the immunosuppressive load; secondly, the increased production of s-Kl by the native non-functioning kidney and/or other extra-renal organs, which may occur during the improvement of graft function. Uremic substances decrease klotho expression through DNA methylation [16]. Thus, improvement in uremia after KT may lead to increased s-Kl production in the native kidney and other klotho-expressing organs. Alternatively, a higher phosphaturia level might induce tubular damage leading to lower klotho expression, as reported by [17]. Indeed, a significantly higher phosphaturia level was observed in the GFR ≤ 40 group due possibly to increased FGF-23 levels. The fact that a better graft function was associated with a higher recovery of s-Kl supports the hypothesis that the post-transplant s-Kl increase could be the result of a combination of a proper renal function after recovering from IRI plus improved klotho expression by the native kidney. Whether this can prolong long-term graft survival is currently undetermined.

Overall, FGF-23 levels declined at month 3 as graft function improved, independently of CIT, and remained reduced throughout the follow-up, as reported by [7]. Nevertheless, a more important drop in FGF-23 levels was evidenced in patients with better graft function. In fact, GFR correlated with FGF-23 levels at months 3 and 24. FGF-23 autoregulates its effect by inhibiting the expression of α-klotho. Thus, increased klotho expression would be expected after the fall in FGF-23 levels at month 3. Interestingly, s-Kl declined in this post-KT period. We speculate that IRI could lead to a state of acute reversible klotho deficiency during the first three months post-KT, even when FGF-23 levels decrease, regardless of CIT. Besides IRI, further risk factors for tubular damage, such as increased phosphaturia, may affect FGF-23 and klotho expression in distal tubules, as reported by [17]. In this line, hypophosphatemia was pronounced in our patients during the first three months after KT, possibly due to inadequate phosphaturia levels over this period from increased pre-KT FGF-23 levels. Indeed, a numerically higher phosphaturia level was observed in our patients with CIT ≥ 14 h. Longitudinal studies are needed to elucidate this concern.

SCI, including borderline lesions and a higher grade of graft inflammation, is very common post-transplantation (≈40–50%) when protocol biopsies are routinely used, even under proper immunosuppression [18,19]. Our results showed a significant positive association between SCI and s-Kl at month 3 post-KT in the multivariate regression analysis. A priori, this result represented a paradox because local and systemic inflammation may downregulate klotho expression through a NFκB-dependent mechanism [4]. However, it is plausible that the positive correlation found between SCI and s-Kl at month 3 post-KT may be an early compensatory response to graft inflammation and that s-Kl would act as an anti-inflammatory molecule. The fact that s-Kl is found to be remarkably high in major inflammatory diseases such as rheumatoid arthritis [20], alcoholism-associated inflammation [21] and sepsis [22] supports this argument. Thus, individual cytokines in the inflammatory environment (e.g., proinflammatory cytokines such as TNF-α) might exert a dual effect on soluble klotho, decreasing the transcription of the transmembrane molecule but increasing the shedding of soluble klotho by augmenting ADAM expression, a secretase that promotes the shedding of the soluble protein klotho [23,24]. Further studies assessing klotho expression in graft tissue samples obtained from protocol biopsies could help to address this hypothesis.

In conclusion, a reduction in s-Kl at month 3 post-KT could be explained by a longer CIT and delayed graft function as well as by impaired graft function. Given the potential negative effect of s-Kl deficiency on accelerated senescence and both graft and patient survival, efforts should be made to decrease CIT, optimize graft function and minimize SCI in the KT population.

## Figures and Tables

**Table 1:** Baseline clinical data.

	*N* = 146
Donor age (years)	56.4 ± 11.6
Expanded criteria donor, *n* (%)	77 (52.7)
Donor after cardiac death, *n* (%)	46 (31.5)
Recipient age (years)	55.9 ± 12.7
Recipient weight (kg)	73.1 ± 14.4
Recipient BMI (kg/m^2^)	22.3 ± 7.3
Male, *n* (%)	93 (63.7)
Prior CVD, *n* (%)	20 (13.7)
Hemodialysis, *n* (%)	120 (82.2)
Current smoker, *n* (%)	18 (12.6)
Cause of ESRD, *n* (%)	
Glomerulonephritis	23 (15.8)
Diabetes	24 (16.4)
ADPKD	25 (17.1)
Interstitial nephropathy	7 (4.8)
Autoimmune	5 (3.4)
Nephrosclerosis	11 (7.5)
Unknown	36 (24.7)
Other	15 (10.3)
Induction therapy, *n* (%)	
None	22 (15.1)
Basiliximab	51 (34.9)
Thymoglobulin	73 (50)
CIT (h)	13.4 ± 3.5
Re-transplantation, *n* (%)	12 (8.2)
DGF, *n* (%)	51 (35.4)
Diabetes, *n* (%)	38 (26)
Hypertension, *n* (%)	125 (85.6)
Maximum PRA (%)	16.7 ± 33.8
Total HLA mismatches (*n*)	6.9 ± 1.7
Previous transfusions (*n*)	0.23 ± 0.42
Serum calcium (mg/dL)	7.9 ± 0.6
Serum phosphate (mg/dL)	5.6 ± 1.6
PTH (pg/mL)	425.1 ± 331.5
Serum klotho levels (pg/mL)	434.5 ± 253.3
Serum FGF-23 levels (pg/mL)	780.6 ± 568.3
Serum TNF levels (pg/mL)	8.8 ± 13.8
Serum TWEAK levels (pg/mL)	437.5 ± 269.6
Vitamin D treatment, *n* (%)	40 (27.4)

Abbreviations: ADPKD, autosomal dominant polycystic kidney disease; BMI, body mass index; CIT, cold ischemia time; CVD, cardiovascular disease; DGF, delayed graft function; ESRD, end-stage renal disease; FGF-23, fibroblast growth factor-23; HLA, human leucocyte antigen; PRA, panel-reactive antibodies; PTH, parathyroid hormone; TNF, tumor necrosis factor; TWEAK, tumor necrosis factor-like weak inducer of apoptosis.

**Table 2:** Characteristics of recipients at month 3 post-kidney transplantation according to the cold ischemia time <14 vs. ≥14 h.

	CIT < 14 h (*n* = 75)	CIT ≥ 14 h (*n* = 71)	*p*
Donor age (years)	56.2 ± 12.4	55.6 ± 13.1	0.786
Expanded criteria donor, *n* (%)	41 (54.7)	36 (50.7)	0.632
Donor after cardiac death, *n* (%)	29 (38.7)	17 (23.9)	0.056
Recipient age (years)	56 ± 12.5	56.7 ± 10.7	0.727
Recipient weight (kg)	71.3 ± 12.5	76.2 ± 19.6	0.079
Recipient BMI (kg/m^2^)	21.5 ± 3.2	23.3 ± 9.9	0.164
Male, *n* (%)	43 (57.3)	50 (70.4)	0.100
Prior CVD, *n* (%)	9 (12)	11 (15.5)	0.540
Hemodialysis, *n* (%)	60 (80)	60 (84.5)	0.328
Current smoker, *n* (%)	6 (8.1)	12 (17.4)	0.246
Cause of ESRD, *n* (%)			0.340
Glomerulonephritis	13 (17.3)	10 (14.1)	
Diabetes	11 (14.7)	13 (18.3)	
ADPKD	13 (17.3)	12 (16.9)	
Interstitial nephropathy	5 (6.7)	2 (2.8)	
Autoimmune	5 (6.7)	0 (0)	
Nephrosclerosis	6 (8)	5 (7)	
Unknown	16 (21.3)	20 (28.2)	
Other	6 (8)	9 (12.7)	
Induction therapy, *n* (%)			0.023
None	8 (10.7)	14 (18.6)	
Basiliximab	21 (28)	30 (42.9)	
Thymoglobulin	46 (61.3)	27 (38.6)	
PTDM, *n* (%)	21 (27.9)	24 (34.2)	0.540
Maximum PRA (%)	17.2 ± 33.9	16.3 ± 33.9	0.871
DGF, *n* (%)	18 (24.3)	33 (47.1)	0.004
Acute rejection, *n* (%)	12 (15.7)	17 (23.9)	0.308
Glycemia (mg/dL)	114.2 ± 69.9	105.2 ± 44.9	0.373
HbA1c (%)	6.03 ± 1.4	6.02 ± 0.9	0.988
Total cholesterol (mg/dL)	175.8 ± 39.1	169.5 ± 42.9	0.400
HDL cholesterol (mg/dL)	55.5 ± 17.4	48.9 ± 15.9	0.035
LDL cholesterol (mg/dL)	91.9 ± 33.2	89.4 ± 29.8	0.677
Triglycerides (mg/dL)	136.1 ± 73.6	152.2 ± 84.6	0.260
Serum calcium (mg/dL)	9.3 ± 0.5	9.3 ± 0.7	0.685
Serum phosphate (mg/dL)	3.2 ± 0.7	3.3 ± 0.7	0.166
FEPhos (%)	31.5 ± 14.4	34.6 ± 16.5	0.300
PTH (pg/mL)	103.8 ± 64.4	114.7 ± 89.1	0.442
Hypertension, *n* (%)	66 (88.0)	59 (83.1)	0.399
SBP (mmHg)	125.7 ± 10.8	128.9 ± 10.9	0.104
DBP (mmHg)	72.4 ± 8.6	73.2 ± 7.8	0.573
Tacrolimus levels (ng/mL)	9.8 ± 3.4	9.8 ± 3.3	0.939
Previous transfusions (*n*)	0.22 ± 0.42	0.25 ± 0.43	0.704
Total HLA mismatches (*n*)	6.9 ± 1.8	7.01 ± 1.7	0.605
Proteinuria (mg/24 h.)	328 ± 324.6	358.6 ± 390.7	0.732
eGFR CKD-EPI (mL/min/1.73 m^2^)	45.5 ± 15.4	40.2 ± 17.2	0.072
ΔCr (mg/dL)	−0.17 ± 0.36	−0.37 ± 0.55	0.014
Serum klotho levels (pg/mL)	420.8 ± 241.8	318.2 ± 215.3	0.052
Serum FGF-23 levels (pg/mL)	174.8 ± 217.5	246.9 ± 371.8	0.340
Serum TNF levels (pg/mL)	7.1 ± 10.7	3.7 ± 5.3	0.096
Serum TWEAK levels (pg/mL)	444 ± 363.7	418.9 ± 218.1	0.738
Vitamin D treatment, *n* (%)	20 (27.8)	20 (29)	0.614

Abbreviations: ADPKD, autosomal dominant polycystic kidney disease; BMI, body mass index; CIT, cold ischemia time; CVD, cardiovascular disease; ΔCr, creatinine delta; DGF, delayed graft function; DBP, diastolic blood pressure; eGFR CKD-EPI, estimated glomerular filtration rate with CKD-EPI formula; ESRD, end-stage renal disease; FEPhos, fractional excretion of phosphorus; FGF-23, fibroblastic growth factor-23; HDL, high-density lipoprotein; HLA, human leucocyte antigen; LDL, low-density lipoprotein; PRA, panel-reactive antibodies; PTDM, post-transplantation diabetes mellitus; PTH, parathyroid hormone; SBP, systolic blood pressure; TNF, tumor necrosis factor; TWEAK, tumor necrosis factor-like weak inducer of apoptosis.

**Table 3:** Characteristics of recipients at month 3 post-kidney transplantation according to the allograft function (GFR in mL/min/1.73 m^2^).

	GFR ≤ 40 (*n* = 55)	GFR > 40 (*n* = 68)	*p*
Donor age (years)	58.7 ± 9.7	53.9 ± 12.1	0.018
Expanded criteria donor, *n* (%)	36 (65.5)	29 (42.6)	0.012
Donor after cardiac death, *n* (%)	16 (29.1)	20 (29.4)	0.969
Recipient age (years)	56.3 ± 11.1	54.4 ± 14	0.426
Recipient weight (kg)	75.5 ± 15.5	72.6 ± 18.5	0.354
Recipient BMI (kg/m^2^)	22.4 ± 4.2	22.5 ± 9.8	0.938
Male, *n* (%)	37 (67.3)	41 (60.3)	0.242
Prior CVD, *n* (%)	5 (9.1)	10 (14.7)	0.344
Hemodialysis, *n* (%)	46 (83.6)	55 (80.9)	0.898
Current smoker, *n* (%)	6 (10.9)	9 (13.4)	0.243
Cause of ESRD, *n* (%)			0.260
Glomerulonephritis	6 (10.9)	11 (16.2)	
Diabetes	14 (25.5)	7 (10.3)	
ADPKD	10 (18.2)	11 (16.2)	
Interstitial nephropathy	1 (1.8)	4 (5.9)	
Autoimmune	1 (1.8)	4 (5.9)	
Nephrosclerosis	5 (9.1)	4 (5.9)	
Unknown	11 (20)	20 (29.4)	
Other	7 (12.7)	7 (10.3)	
Induction therapy, *n* (%)			0.165
None	5 (9.1)	11 (16.4)	
Basiliximab	24 (43.6)	19 (28.4)	
Thymoglobulin	26 (47.3)	38 (55.2)	
CIT (h)	14 ± 3.5	13.1 ± 3.5	0.141
PTDM, *n* (%)	19 (34.5)	20 (30)	0.680
DGF, *n* (%)	28 (50.9)	16 (23.5)	0.002
Acute rejection, *n* (%)	17 (30)	6 (8.3)	0.009
Maximum PRA (%)	13.9 ± 29.7	20.4 ± 38.3	0.302
Glycemia (mg/dL)	110.5 ± 57.8	105.3 ± 52.2	0.601
HbA1c (%)	6.3 ± 1.4	5.8 ± 0.9	0.041
Total cholesterol (mg/dL)	169.1 ± 39.5	175.8 ± 42.2	0.398
HDL cholesterol (mg/dL)	49.1 ± 18.6	56 ± 15.9	0.042
LDL cholesterol (mg/dL)	85 ± 28.8	94.1 ± 33.7	0.148
Triglycerides (mg/dL)	156.7 ± 95.3	134.7 ± 62.1	0.161
Serum calcium (mg/dL)	9.2 ± 0.7	9.3 ± 0.5	0.836
Serum phosphate (mg/dL)	3.3 ± 0.8	3.2 ± 0.6	0.320
FEPhos (%)	28.6 ± 12.4	40.4 ± 16.6	<0.001
PTH (pg/mL)	117.7 ± 96.3	101.0 ± 58.9	0.270
Hypertension, *n* (%)	45 (81.8)	60 (88.2)	0.317
SBP (mmHg)	129.5 ± 10.4	125.8 ± 11.6	0.093
DBP (mmHg)	73.8 ± 8.9	72.3 ± 7.9	0.352
Tacrolimus levels (ng/mL)	9.9 ± 3.8	9.7 ± 3.1	0.808
Previous transfusions (*n*)	0.24 ± 0.43	0.28 ± 0.45	0.658
Total HLA mismatches (*n*)	6.96 ± 1.8	6.97 ± 1.6	0.982
Proteinuria (mg/24 h.)	422.9 ± 508.6	256.2 ± 145.3	0.124
eGFR CKD-EPI (mL/min/1.73 m^2^)	29.2 ± 7.8	53.9 ± 12.9	<0.001
Serum klotho levels (pg/mL)	304.9 ± 180.4	398.1 ± 244.8	0.079
Serum FGF-23 levels (pg/mL)	335.2 ± 422.9	121.4 ± 117	0.018
Serum TNF levels (pg/mL)	5.1 ± 6.2	5.9 ± 10.7	0.716
Serum TWEAK levels (pg/mL)	425.9 ± 338.3	445.9 ± 285.6	0.796
Vitamin D treatment, *n* (%)	18 (33.3)	16 (23.9)	0.255

Abbreviations: ADPKD, autosomal dominant polycystic kidney disease; BMI, body mass index; CIT, cold ischemia time; CVD, cardiovascular disease; DBP, diastolic blood pressure; DGF, delayed graft function; eGFR CKD-EPI, estimated glomerular filtration rate with CKD-EPI formula; ESRD, end-stage renal disease; FEPhos, fractional excretion of phosphorus; FGF-23, fibroblastic growth factor-23; HDL, high-density lipoprotein; HLA, human leukocyte antigen; LDL, low-density lipoprotein; PRA, panel-reactive antibodies; PTDM, post-transplantation diabetes mellitus; PTH, parathyroid hormone; SBP, systolic blood pressure; TNF, tumor necrosis factor; TWEAK, tumor necrosis factor-like weak inducer of apoptosis.

**Table 4:** Banff scores in the baseline protocol biopsy at 3 months post-transplant.

	NI (*n* = 32)	SCI (*N* = 65)	*p*
g (0–3)	0.06 ± 0.35	0.23 ± 0.49	0.058
ptc (0–3)	0.38 ± 0.61	0.69 ± 0.66	0.028
t (0–3)	0.22 ± 0.42	1.34 ± 0.57	<0.001
i (0–3)	0.22 ± 0.61	1.26 ± 0.48	<0.001
v (0–3)	0	0.11 ± 0.48	0.070
ci (0–3)	0.34 ± 0.48	0.48 ± 0.62	0.248
ct (0–3)	0.31 ± 0.47	0.48 ± 0.62	0.149
cg (0–3)	0	0	-
cv (0–3)	0.75 ± 0.84	0.63 ± 0.82	0.507
ah (0–3)	0.34 ± 0.55	0.57 ± 0.75	0.096
mm (0–3)	0.06 ± 0.25	0.15 ± 0.51	0.235
IFTA (0–3)	0.22 ± 0.71	0.35 ± 0.72	0.383
ct + ci	0.66 ± 0.94	0.95 ± 1.22	0.188
ct + ci + cg + cv	1.41 ± 1.46	1.59 ± 1.64	0.603
Tacrolimus levels (ng/mL)	9.73 ± 3.16	9.41 ± 3.31	0.658
Serum klotho levels (pg/mL)	351.4 ± 210.4	386.2 ± 247.1	0.529
FGF-23 levels (pg/mL)	122.7 ± 63.1	197.4 ± 172.3	0.055

Abbreviations: ah, arteriolar hyaline thickening; ci, chronic interstitial fibrosis; cg, transplant glomerulopathy; ct, chronic tubular; cv, fibrous intimal thickening; FGF-23, fibroblastic growth factor-23; g, glomerulitis; i, interstitial infiltration; mm, mesangial matrix expansion; NI, no inflammation; ptc, peritubular capillaritis; t, tubulitis; v, arteritis; IFTA: proportion of patients with sum of interstitial fibrosis and tubular atrophy ≥ 2.

**Table 5:** Multivariate linear regression analysis of factors associated with s-Kl at three months post-transplant.

Variable	Beta	Standardized Beta Coefficient	95% CI	*p* Value
CIT (<14 h vs. ≥14 h)	−110.386	−0.225	−196.018–(−24.755)	0.012
Basal s-Kl (pg/mL)	0.641	0.651	0.473–0.809	0.000
Recipient age (y)	1.708	0.084	−1.755–5.171	0.328
ECD (%)	−66.998	−0.135	−157.093–23.097	0.142
SCI (%)	113.087	0.212	22.770−203.404	0.015
eGFR (>40 vs. ≤40 mL/min/1.73 m^2^)	95.833	0.195	5.632−186.035	0.038
FEPhos (%)	2.062	0.129	−1.068–5.192	0.193
Proteinuria (mg/24 h)	0.043	0.050	−0.106–0.191	0.570

Abbreviations: CIT, cold ischemia time; ECD, expanded criteria donors; s-Kl, serum klotho levels; FEPhos, fractional excretion of phosphorus; GFR, estimated glomerular filtration rate; SCI, subclinical inflammation.

## Data Availability

The data presented in this study are available on request from the corresponding author. In compliance with Spanish Organic Law 15/1999, the data are not publicly available.
